# The Pathogenic Role of Long Non-coding RNA H19 in Atherosclerosis *via* the miR-146a-5p/ANGPTL4 Pathway

**DOI:** 10.3389/fcvm.2021.770163

**Published:** 2021-11-08

**Authors:** Shi-Feng Huang, Guifang Zhao, Xiao-Fei Peng, Wen-Chu Ye

**Affiliations:** ^1^Qingyuan People's Hospital, The Sixth Affiliated Hospital of Guangzhou Medical University, Qingyuan, China; ^2^Department of General Surgery, Qingyuan People's Hospital, The Sixth Affiliated Hospital of Guangzhou Medical University, Qingyuan, China

**Keywords:** lncRNA H19, atherosclerosis, miR-146a-5p, ANGPTL4, lipid accumulation

## Abstract

The abnormally expressed long non-coding RNA (lncRNA) H19 has a crucial function in the development and progression of cardiovascular disease; however, its role in atherosclerosis is yet to be known. We aimed to examine the impacts of lncRNA H19 on atherogenesis as well as the involved mechanism. The outcomes from this research illustrated that the expression of lncRNA H19 was elevated in mouse blood and aorta with lipid-loaded macrophages and atherosclerosis. Adeno-associated virus (AAV)-mediated lncRNA H19 overexpression significantly increased the atherosclerotic plaque area in apoE^−/−^ mice supplied with a Western diet. The upregulation of lncRNA H19 decreased the miR-146a-5p expression but increased the levels of ANGPTL4 in mouse blood and aorta and THP-1 cells. Furthermore, lncRNA H19 overexpression promoted lipid accumulation in oxidized low-density lipoprotein (ox-LDL)-induced THP-1 macrophages. However, the knockdown of lncRNA H19 served as a protection against atherosclerosis in apoE^−/−^ mice and lowered the accumulation of lipids in ox-LDL-induced THP-1 macrophages. lncRNA H19 promoted the expression of ANGPTL4 *via* competitively binding to miR-146a-5p, thus promoting lipid accumulation in atherosclerosis. These findings altogether demonstrated that lncRNA H19 facilitated the accumulation of lipid in macrophages and aggravated the progression of atherosclerosis through the miR-146a-5p/ANGPTL4 pathway. Targeting lncRNA H19 might be an auspicious therapeutic approach for preventing and treating atherosclerotic disease.

## Introduction

Atherosclerosis has been known to be characterized by lipid accumulation in the intima of the arteries, which forms the pathological mechanism of cardiovascular diseases (CVDs), such as atrial fibrillation, heart failure, and coronary artery disease (CAD) (1). During atherogenesis, macrophages uptake the modified lipoproteins to enhance endothelial permeability and increase foam cell formation, a hallmark of atherosclerotic plaques (2). Angiopoietin-like 4 (ANGPTL4), which is a released glycoprotein, regulates lipid metabolism and insulin sensitivity (3, 4). ANGPTL4 has been recognized as a powerful suppressor of lipoprotein lipase (LPL) *in vitro*. The overexpression of ANGPTL4 increased the plasma triglyceride levels, whereas the knockdown of ANGPTL4 decreased the plasma triglyceride levels in mice (5). ANGPTL4 is directly associated with the risk of atherosclerosis and type 2 diabetes mellitus (T2DM) (6). Thus, it is critical to comprehend the molecular mechanisms involved in ANGPTL4 regulation for the development of new therapeutic interventions for atherosclerosis.

Long non-coding RNAs (lncRNAs), which are non-protein-coding transcripts surpassing 200 nucleotides in length, have a vital function in regulating gene expression (7, 8). Aberrant expression of lncRNA is strongly correlated with lipid accumulation and atherosclerosis (9–12). LncRNA H19, located on chromosome 11p15.5 in humans, is a canonical paternally imprinted and maternally expressed gene (13). lncRNA H19 levels detected in peripheral blood monocyte cells (PBMCs) obtained from CAD patients are higher than those observed in PBMCs isolated from healthy individuals (14). Importantly, the knockdown of lncRNA H19 has been demonstrated to alleviate atherosclerosis (15); however, the overexpression of lncRNA H19 contributed to the progression of atherosclerosis and induced ischemic stroke (16). Although lncRNA H19 facilitates the development as well as the progression of atherosclerosis, the participation of lncRNA H19 in regulating excessive lipid-induced atherosclerosis remains elusive.

MiRNAs (miRNAs) are small non-coding RNAs that function as negative regulators of gene expression through the gene silencing (17). Recently, a growing number of studies have been focused on the roles of miRNAs in various cellular functions (18). Notably, an individual miRNA can have hundreds of targets, while a single target gene may be regulated by many different miRNAs. Moreover, increasing studies demonstrated expression of multiple miRNAs is altered in atherosclerosis, implying that miRNAs may play important roles in the development of atherosclerosis (19, 20). MicroRNA-146a-5p (miR-146a-5p) is located in homo sapiens chromosome 5q33.3 and correlates with metabolic diseases (21, 22). Numerous lncRNAs can be combined with the specific miRNAs to competitively upregulate the expression of target genes, called competing endogenous RNAs (ceRNAs) (23). A recent study showed that lncRNA H19 functions as a ceRNA to facilitate the occurrence and development of atherosclerosis (16, 24, 25). However, whether lncRNA H19 sponges miR-146a-5p to regulate ANGPTL4 expression needs to be addressed.

In this research, we discovered that the lncRNA H19 expression was dramatically upregulated in the blood and aortic tissues of apoE^−/−^ mice and oxidized low-density lipoprotein (ox-LDL)-treated THP-1 cells. Subsequent overexpression of lncRNA H19 *in vivo* demonstrated that lncRNA H19 overexpression significantly increased the progression of atherosclerosis. Furthermore, lncRNA H19 served as a microRNA (miRNA) sponge in the positive regulation of the ANGPTL4 expression by sponging miR-146a-5p, thereby promoting lipid accumulation in atherosclerosis. Thus, our research offers novel acumens into the molecular role of lncRNA H19/miR-146a-5p/ANGPTL4 signaling pathway in lipid accumulation and highlights lncRNA H19 as a promising therapeutic target for atherosclerosis.

## Materials and Methods

### Animal Modeling

Approval of all the animal experimentation was gotten from the Animal Care and Use Committee at the Sixth Affiliated Hospital of Guangzhou Medical University (ethics number: LAEC-201910-002). This study was conducted only with males, as it is well-known that atherosclerotic plaques develop more reproducibly and with less biological variability. Male apolipoprotein E deficient (apoE)^−/−^ mice (weight, 20–25 g; age, 4 weeks) on a C57BL/6J genetic background were obtained from Nanjing Junke Biological Engineering Co. Ltd. (Nanjing, China). A total of 24 apoE^−/−^ mice and 6 wild-type C57BL/6 mice (weight: 20–25 g; age: 4 weeks) were used. Following 1 week of adaptive feeding, the initiation of the experimental modeling was done. The mice were housed under a regular cycle involving 12-h of light and 12 h of darkness. The humidity was maintained at 60% and the temperature was controlled at 22°C. The mice were allowed unrestricted access to food and drinking water.

### Transfection With Adeno-Associated Virus

According to the transfection scheme, 24 apoE^−/−^ mice were randomly allocated to four cohorts, namely, adeno-associated virus (AAV, JiKai Gene, Shanghai, China)-sh-NC, AAV-sh-H19, AAV-OE-NC, and AAV-OE-H19 groups (*n* = 6). Male apoE^−/−^ mice were treated with either the AAV-sh-NC, AAV-sh-H19, AAV-OE-NC, or AAV-OE-H19 *via* tail vein at a titer of 1.0 × 10^11^ vector genomes/ml. All cohorts were treated with tail vein injection of the corresponding AAV; all AAV suspensions were of the same titer and dosage. Male apoE^−/−^ mice were supplied with a high-fat diet containing 1.25% cholesterol (HFD, D12108C, Research Diets, New Brunswick, NJ, USA) for 1 week and subsequently treated with AAV scrambled small hairpin RNA (AAV-sh-NC), AAV-sh-H19, AAV-OE-NC, and AAV-OE-H19; the mice were continually supplied with HFD for 8 weeks. During the construction of an atherosclerosis model, AAV was injected at 5, 8, and 12 weeks. After 8 weeks rearing, male apoE^−/−^ mice were fasted for 6 h, then placed under isoflurane anesthesia (RWD, Shenzhen, China). Anesthetic used was 1% isoflurane, with the dosage depended on the animal's body weight (40 mg/kg). Blood samples were obtained from the retro-orbital venous plexus using a capillary tube. The mice were then sacrificed by cervical dislocation and organic tissues collected for the following experimental analyses. These analyses were performed blinded to which groups the animals were allocated.

### Evaluation of en Face Lesion Area

After euthanizing the apoE^−/−^ mice, the entire aorta tissues were obtained, followed by the removal of the adventitial tissues. Subsequently, the aorta was opened and oil red O (Sigma, St. Louis, MO, USA) was utilized for staining. Next, the aortal was photographed using a stereomicroscope (Zeiss, Jena, Germany). The proportion of lesion area that stained after application of oil red O on the surface of the aorta was estimated utilizing the Image-Pro Plus 7.0 software (Media Cybernetics, Rockville, MD, USA).

### Evaluation of Atherosclerotic Lesions in the Aortic Root

The mice were sacrificed through exsanguination under anesthesia, and subsequently, the upper part of their hearts was separated from the proximal aorta. After washing using phosphate-buffered saline (PBS), the hearts were entrenched in an optimal cutting temperature (OCT) compound (Sakura, Tokyo, Japan) and frozen. A cryostat microtome was used to prepare serial 6-μm-thick cryosections of the 3 aortic valves, and these sections were kept on glass slides. Then, oil red O, hematoxylin–eosin (HE) and Masson staining were subsequently carried out (26). The Image J software (NIH Image J system, Bethesda, MD) was utilized for quantitative analysis.

### Cell Culture and Lentiviral Vector Transfection

THP-1 cells were procured from the American Type Culture Collection (ATCC: TIB 202, USA). Culturing of the THP-1 cells was done in the Roswell Park Memorial Institute (RPMI) 1640 medium (Thermo Fisher Scientific, USA) comprising 10% fetal bovine serum (FBS) in a CO_2_ concentration of 5% and a temperature of 37°C. Subsequently, differentiation of THP-1 cells into macrophages was done after treating them with 80 nM of phorbol-12-myristate-13-acetate (PMA; MCE, China) for 48 h. The lentivirus used was purchased from Shanghai Jikai Gene Chemical Technology Co., Ltd. (Shanghai, China). THP-1 macrophages were transduced using the lentivirus supernatants (LV-NC, LV-H19, LV-sh-NC, or LV-sh-H19) with 8 μg/ml of polybrene (Sigma–Aldrich, St Louis, MO, USA). After 24 h of incubation, THP-1 macrophages were maintained in a complete medium (10% FBS and RPMI 1640). Real-time reverse transcription polymerase chain reaction (qRT-PCR) was performed to confirm the transfection efficiency.

### miR-146a-5p Mimic/Inhibitor Transfection and ANGPTL4 Knockdown

The miR-146a-5p mimic, miR-146a-5p inhibitor as well as mimic/inhibitor negative control (mimic/inhibitor NC) were procured from GenePharma (Suzhou, China). Transfection of THP-1 cells was done with miR-146a-5p mimic/inhibitor or their negative controls using the lipofectamine 3000 reagent (Invitrogen, CA, USA) for 48 h as per the instructions stipulated in the kit. Subsequently, the transfection efficiency was measured using qRT-PCR. Both small interfering RNA (siRNA) targeting ANGPTL4 (si-ANGPTL4) and non-silencing siRNA were designed and synthesized by GenePharma. Transfection of both siRNAs (80 nM) into THP-1 cells was done utilizing the lipofectamine 3000 reagent for a duration of 24 h. qRT-PCR, as well as western blot (WB), were carried out to assess the efficacy of ANGPTL4 knockdown.

### Bioinformatics Prediction and Luciferase Reporter Assay

The online datasets such as starBase 2.0 and BiBiServ2-RNAhybrid (https://bibiserv.cebitec.uni-bielefeld.de/rnahybrid/) were utilized to forecast the interplay between miR-146a-5p and ANGPTL4 or lncRNA H19. The HEK-293T cells were added to a 24-well plate at a concentration of 2.0 × 10^4^/well. The sequences of lncRNA H19 and ANGPTL4 non-coding sequence [3′ untranslated region (3′-UTR)] comprising the putative miR-146a-5p binding site were amplified using PCR and subsequently cloned downstream of the pGL3 firefly luciferase reporter plasmids (Promega, WI, USA) labeled as lncRNA H19-WT and ANGPTL4-WT. Mutation of the putative miR-146a-5p binding sites was done to produce the matching mutant plasmids (lncRNA H19-Mut and ANGPTL4-Mut). Then, co-transfection of HEK-293T cells with the plasmids and miR-146a-5p mimic was done utilizing the lipofectamine 3000 reagent for a duration of 48 h. Subsequently, the detection of the luciferase activity was done utilizing the dual-luciferase reporter assay system (Promega) followed by the normalization of the firefly luciferase activity to the Renilla luciferase activity.

### RNA Fluorescent *in situ* Hybridization

RNA fluorescence *in situ* hybridization (RNA-FISH) for lncRNA H19 was performed on mouse aortic root tissue and THP-1 cells. Cyanine 3 (Cy3)-labeled lncRNA H19 (human lncRNA 19: 5′-GCTGCTGTTCCGATGGTGTCTTTGATGTTGGGC-3′; mice lncRNA H19: 5′-CAGTTGCCCTCAGACGGAGATGGACG-3′) and 4′,6-diamidino-2-phenylindole (DAPI)-labeled U6 probes were gotten from Servicebio (Guangzhou, China). THP-1 cells were seeded on poly-L-lysine coated slides (Sigma-Aldrich) and fixed in 4% paraformaldehyde for 10 min, washed twice in cold PBS and permeabilized with 70% EtOH. Next, the RNA FISH assays were carried out utilizing a FISH kit (Servicebio, Guangzhou, China) as per the instructions stipulated by the manufacturer.

### RNA Isolation and qRT-PCR

The isolation of the total RNA from the collected blood, tissues as well as cultured THP-1 cells was done utilizing the Blood RNA Isolation Kit (Thermo Fisher Scientific, CA, USA) and TRIzol reagent (Invitrogen) (27). Extracted RNA was quantified for purity and concentration using a NanoDrop 3000 spectrophotometer (Thermo Fisher Scientific). Subsequently, 2 μg of RNA was employed as a template to synthesize complementary DNA (cDNA; Takara, Kyoto, Japan), and qRT-PCR was carried out utilizing the SYBR Green kit (Takara) on a Bio-Rad CFX96 instrument (Bio-Rad, USA) for 32 cycles (95°C for 3 min, 95°C for 15 s and 60°C for 1 min). The U6 was employed as an internal control for glyceraldehyde 3-phosphate dehydrogenase (GAPDH), miR-146a-5p, and other genes. The primers were synthesized by Tianyi Huiyuan Biotech Co., Ltd. (Wuhan, China); they are listed in Table 1. The relative gene expression was analyzed utilizing the 2^−ΔΔCT^ method.

**Table 1:** Sequences used to regulate endogenous miR-146a-5p.

**Gene**	**Forward sequence (5′-3′)**	**Reverse sequence (5′-3′)**
**Human gene**
lncRNA H19	CAGTGGACTTGGTGACGCTGTATG	CGCCTCGCCTAGTCTGGTCTC
miR-146a-5p	ACACTCCAGCTGGGTGAGAACTGAATTCCA	TGGTGTCGTGGAGTCG
ANGPTL4	CAGTCCTCGCACCTGGAA	GCCAGGACATTCATCTCGTC
U6	CTCGCTTCGGCAGCACA	AACGCTTCACGAATTTGCGT
GAPDH	CCTCAAGATCATCAGCAATGCC	TGGTCATGAGTCCTTCCACGAT
**Mouse gene**
lncRNA H19	ACTGGAGACTAGGCCAGGTC	TGGTGTTCAAGAAGGCTGGA
miR-146a-5p	TGAGAACTGAATTCCATGGGTT	TGGTGTCGTGGAGTCG
ANGPTL4	AGAGTTTGCAGACTCAGCTC	CAAGAGGTCTATCTGGCTCT
U6	CTCGCTTCGGCAGCACATATACT	ACGCTTCACGAATTTGCGTGTC
GAPDH	CAGTGCCAGCCTCGTCTCAT	AGGGGCCATCCACAGTCTTC

### Western Blot Analysis

Lysing of the THP-1 cells and tissues was done utilizing the radioimmunoprecipitation assay (RIPA) buffer (Dingguo, Beijing, China) containing 1 mM of dithiothreitol (DTT) and 1 mM of protease inhibitor [phenylmethylsulfonyl fluoride (PMSF)]. The quantification of the protein extracts was done utilizing a bicinchoninic acid (BCA) assay kit (Beyotime, Shanghai, China) as per the instructions stipulated by the manufacturer. Subsequently the protein was subjected to sodium dodecyl sulfate-polyacrylamide gel electrophoresis (SDS-PAGE). This was followed by immunoblotting using the following antibodies: ANGPTL4 (abs136271, Absin, China) and GAPDH (D16H11, Cell Signaling Technology, USA). Washing of the membranes was done utilizing TBST (tris buffered saline and 0.1% Tween 20) followed by incubation using horseradish peroxidase (HRP)-labeled secondary antibodies (1:3,000, Cell Signaling Technology, USA). Visualization of the proteins was done utilizing the BeyoECL Plus kit (Beyotime), and GAPDH was employed as an internal control.

### Oil Red O Staining

Fixing of the THP-1 macrophages was done using 4% paraformaldehyde for a duration of 10 min followed by a 3-times wash with PBS. Subsequently, the oil red O solution was utilized to stain the THP-1 cells for a duration of 20 min and afterward destained using 60% isopropanol for 10 s. A microscope (Olympus BX50) was utilized to capture the pictures of positively stained cells (red) at a magnification of 200×.

### Statistical Analysis

Statistical analyses were articulated as mean ± standard deviation (SD) of a minimum of 3 separate experiments. Comparison between the control cohort and the other cohorts was done utilizing the one-way or two-way analysis of variance (ANOVA). Differences between the 2 cohorts were contrasted utilizing the two-tailed Student's *t*-test. Statistical analysis was executed utilizing the GraphPad Prism 8.0 (GraphPad Prism Software, San Diego, CA, USA) and SPSS 19.0 statistical software (SPSS, Chicago, IL, USA). Statistical significance was delineated as ^*^P < 0.05, ^**^P < 0.01, and ^***^P < 0.001.

## Results

### lncRNA H19 Was Increasingly Expressed in apoE^–/–^ Mice and ox-LDL-Treated THP-1 Cells

**Figure 1:**
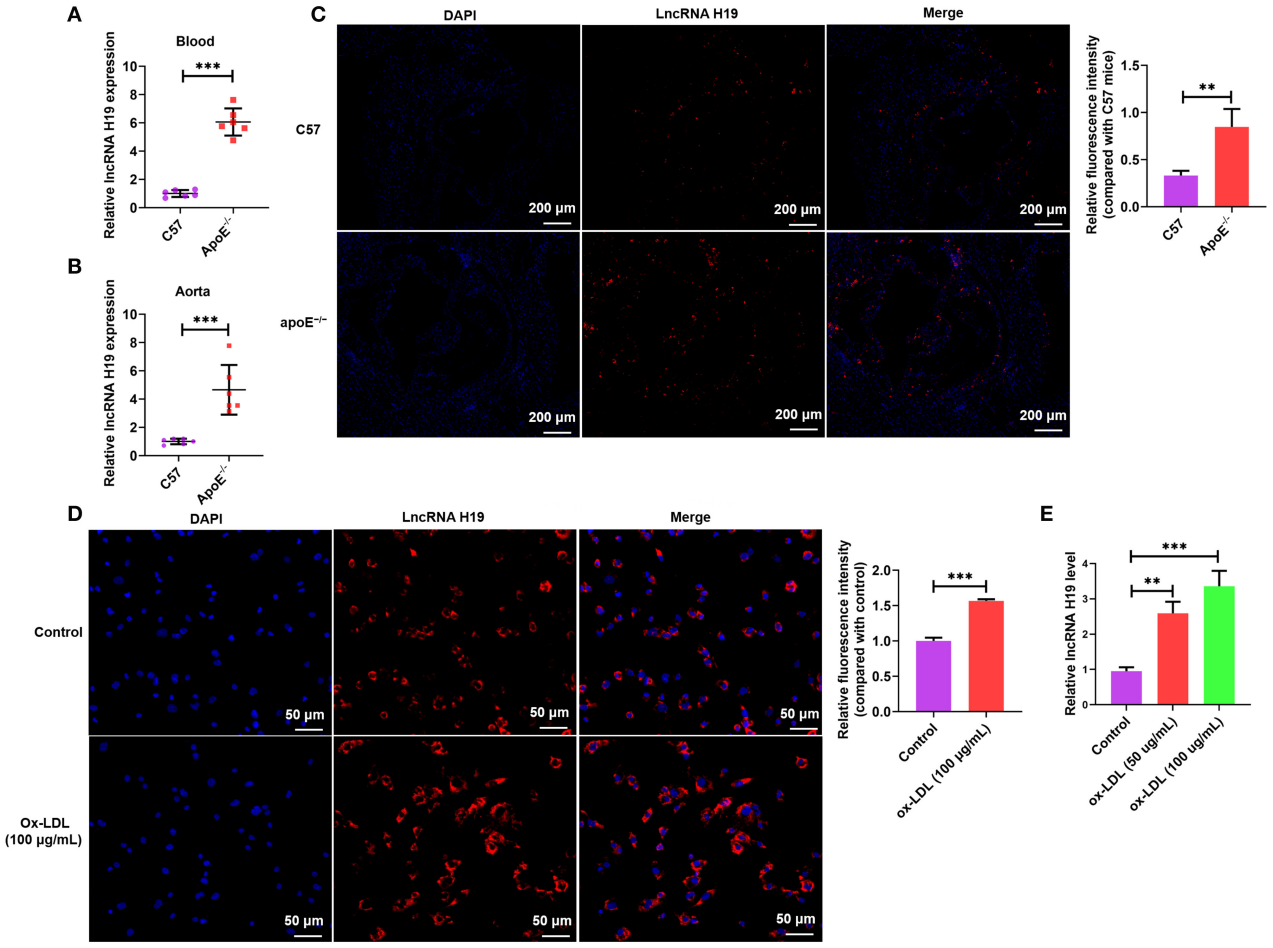
LncRNA H19 was increasingly expressed in the atherosclerotic plaques of apoE^−/−^ mice and macrophages after ox-LDL induction. **(A,B)** lncRNA H19 expression levels in the serum and aorta of apoE^−/−^ and C57BL/6 mice were measured using qRT-PCR. **(C)** FISH analysis of the aorta roots in apoE^−/−^ and C57BL/6 mice. Quantification of lncRNA H19 expression level in the aorta of apoE^−/−^ and C57BL/6 mice. **(D)** The subcellular localization and expression level of lncRNA H19 in ox-LDL-stimulated THP-1-derived macrophages were detected using FISH (200×). Quantification of lncRNA H19 expression level. **(E)** THP-1 cells were treated with ox-LDL (50 and 100 μg/mL) or their controls for 24 h. The lncRNA H19 expression was detected utilizing RT-PCR. The experiment was repeated thrice independently. Data are represented as mean ± SD. ^**^P < 0.01, and ^***^P < 0.001 vs. the control cohort.

Contemporary research reports have demonstrated that aberrant expression and functions of lncRNAs participate in the occurrence as well as the development of various illnesses (28–30), especially atherosclerosis. Recently, lncRNA H19 has been reported as a well-recognized lncRNA linked to the development of atherosclerosis and could be a probable treatment target for atherosclerosis (6, 16). To determine whether lncRNA H19 expression was changed during atherosclerosis, we initially analyzed its expression in the whole blood and aortic tissues of apoE^−/−^ mice using qRT-PCR and RNA-FISH. As illustrated in Figures 1A–C, the expression of lncRNA H19 expression was elevated in apoE^−/−^ mice supplied with a Western diet as opposed to that in the wild-type C57BL/6 mice. The levels of lncRNA H19 were considerably elevated in ox-LDL-treated THP-1 cells as opposed to the control cells (Figures 1D,E). These findings suggested that lncRNA H19 may be an important cause of atherosclerosis.

### lncRNA H19 Facilitated Lipid Accumulation in ox-LDL-Treated THP-1 Cells

**Figure 2:**
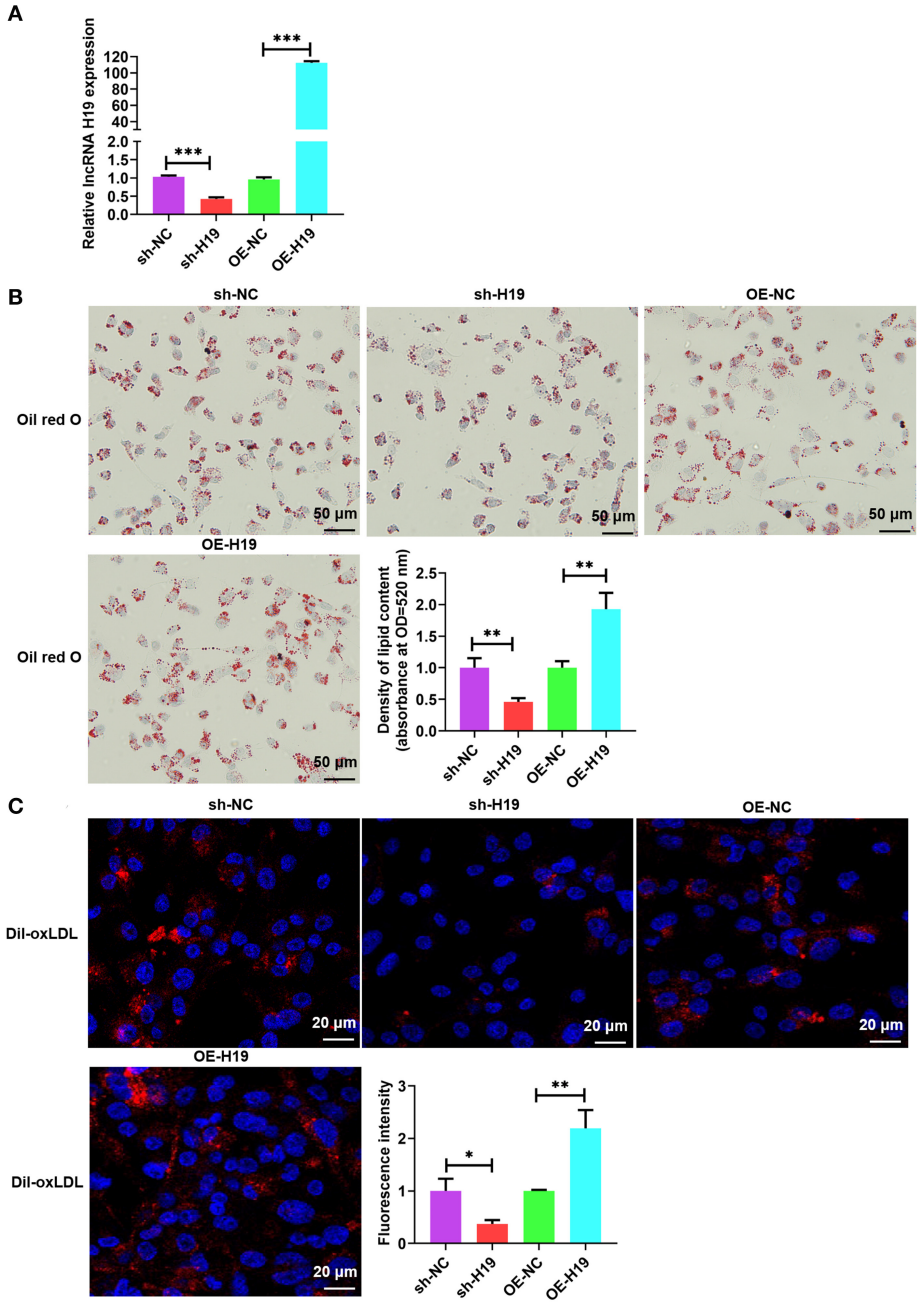
lncRNA H19 facilitated lipid accumulation in ox-LDL-treated THP-1 cells. **(A–C)** THP-1 cells were transfected with sh-NC, sh-H19, OE-NC, or OE-H19 for 48 h. **(A)** lncRNA H19 expression was determined using RT-PCR (*n* = 3). **(B)** Oil red O-stained cells were treated with ox-LDL (100 μg/mL) and evaluated after lncRNA H19 knockdown or overexpression. Typical images of oil red O staining results (200×). Isopropanol extraction was performed for oil red O staining. Oil red O absorbance after isopropanol extraction was measured by quantifying lipids. Illustrative images of oil red O staining (200×). Scale bar = 50 μm. **(C)** Illustrative images demonstrating Dil-ox-LDL uptake after incubation for 1 h. The calculation of the fluorescence intensity was done utilizing Image J. Data are represented as mean ± SD of 3 separate experiments. ^*^P < 0.05, ^**^P < 0.01, and ^***^P < 0.001 vs. the control cohort.

Atherosclerotic plaque is characterized by the accumulation of lipid as well as the formation of lipid-laden foam cells (9). It is well-known that lncRNAs facilitate lipid accumulation in multiple cells (31, 32). To determine the effects of lncRNA H19 on ox-LDL-treated THP-1 cells, lncRNA H19 was knocked down or overexpressed in THP-1 cells infected with lentivirus-mediated lncRNA H19 knockdown or overexpression. The results indicated that the knockdown of lncRNA H19 significantly decreased lncRNA H19 levels; however, the overexpression of lncRNA H19 markedly increased the levels (Figure 2A). To examine the effects of lncRNA H19 on the accumulation of lipid in ox-LDL-treated THP-1 cells, lncRNA H19 was knocked down or overexpressed in THP-1 cells. It was discovered that ox-LDL stimulation-induced foam cell formation (33), and the knockdown of lncRNA H19 significantly inhibited foam cell formation and lipid accumulation, whereas the overexpression of lncRNA H19 promoted lipid accumulation in macrophage foam cells (Figures 2B,C). These observations suggested that lncRNA H19 promoted the formation of foam cells in ox-LDL-treated THP-1 cells, implying that it aggravated the progression of atherosclerosis.

### lncRNA H19 Facilitated the Progression of Atherosclerosis in apoE^–/–^ Mice

**Figure 3:**
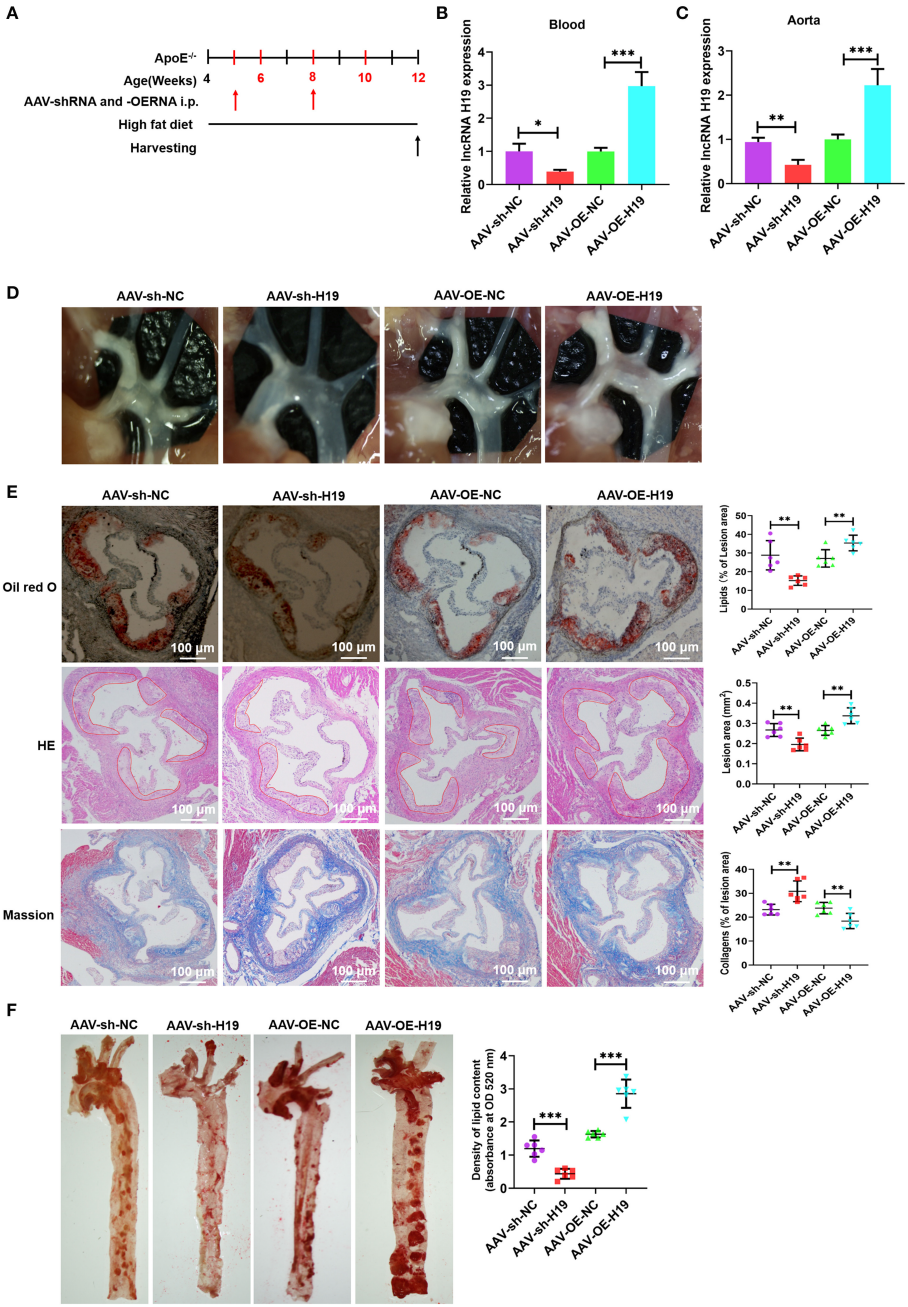
LncRNA H19 aggravates atherosclerosis in apoE^−/−^ mice. **(A)** Flow chart demonstrating the experimental protocol utilized in the *in vivo* experiments. **(B,C)** RT-PCR analysis of lncRNA H19 expression in the blood and aorta of atherosclerotic lesions of AAV-sh-NC-, AAV-sh-H19-, AAV-OE-NC-, and AAV-OE-H19-treated apoE^−/−^ mice. **(D)** Plaques in the aortic arch of apoE^−/−^ mice observed using a stereoscopic microscope. **(E)** Parts of the aortic root was stained with Mason, oil red O, or HE. The lesion area and proportion were computed utilizing the Image J software (*n* = 6). Scale bar = 100 μm. **(F)** The whole aorta was stained with oil red O, and the area of atherosclerotic lesion was computed by analyzing oil red O absorbance after isopropanol extraction (*n* = 6). Data are represented as mean ± SD of 3 separate experiments. ^*^P < 0.05, ^**^P < 0.01, and ^***^P < 0.001 vs. the control cohort.

To evaluate the role and treatment prospective of lncRNA H19 *in vivo*, male apoE^−/−^ mice (weight, 20–25 g; age, 4 weeks) were supplied with a high-fat diet (HFD) for 1 week and subsequently treated with AAV scrambled small hairpin RNA (AAV-sh-NC), AAV-sh-H19, AAV-OE-NC, and AAV-OE-H19; the mice were continually supplied with HFD for 8 weeks (Figure 3A). Foremost, we ascertained the efficiency of lncRNA H19 suppression and overexpression by assessing lncRNA H19 expression in blood and the aorta after treating the mice for 8 weeks. The levels of lncRNA H19 expression identified utilizing qRT-PCR were considerably reduced in the AAV-sh-H19 cohort and elevated in the AAV-OE-H19 cohort as opposed to the matching control cohort (AAV-sh-NC or AAV-OE-NC) (Figures 3B,C). After 8 weeks of feeding HFD to apoE^−/−^ mice, we found a marked reduction in the number as well as the size of atherosclerotic lesions in the aortic arch sections (Figure 3D) in the AAV-sh-H19 cohort as compared with the AAV-sh-NC cohort; however, lncRNA H19 overexpression in the AAV-OE-H19 cohort significantly increased plaque formation in the aortic arch regions (Figure 3D) compared with the AAV-OE-NC cohort. Oil red O, and HE staining of the aortic root cross-sections illustrated that the overexpression of lncRNA H19 also considerably increased the lesion area, enhanced lipid deposition, and lowered collagen content (Figure 3E). The knockdown of lncRNA H19 decreased the lesion area, suppressed deposition of lipids, and elevated collagen content as demonstrated by Masson, oil red O, and HE staining (Figure 3E). Moreover, lncRNA H19 overexpression increased the en face lesions in the aorta (Figure 3F), which did not occur when lncRNA H19 was knocked down (Figure 3F). These *in vivo* findings collectively illustrate that lncRNA H19 serves as a vital participant in the progression of atherosclerosis.

### lncRNA H19 Inhibited miR-146a-5p Expression by Acting as a Sponge in THP-1 Macrophage-Derived Foam Cells

**Figure 4:**
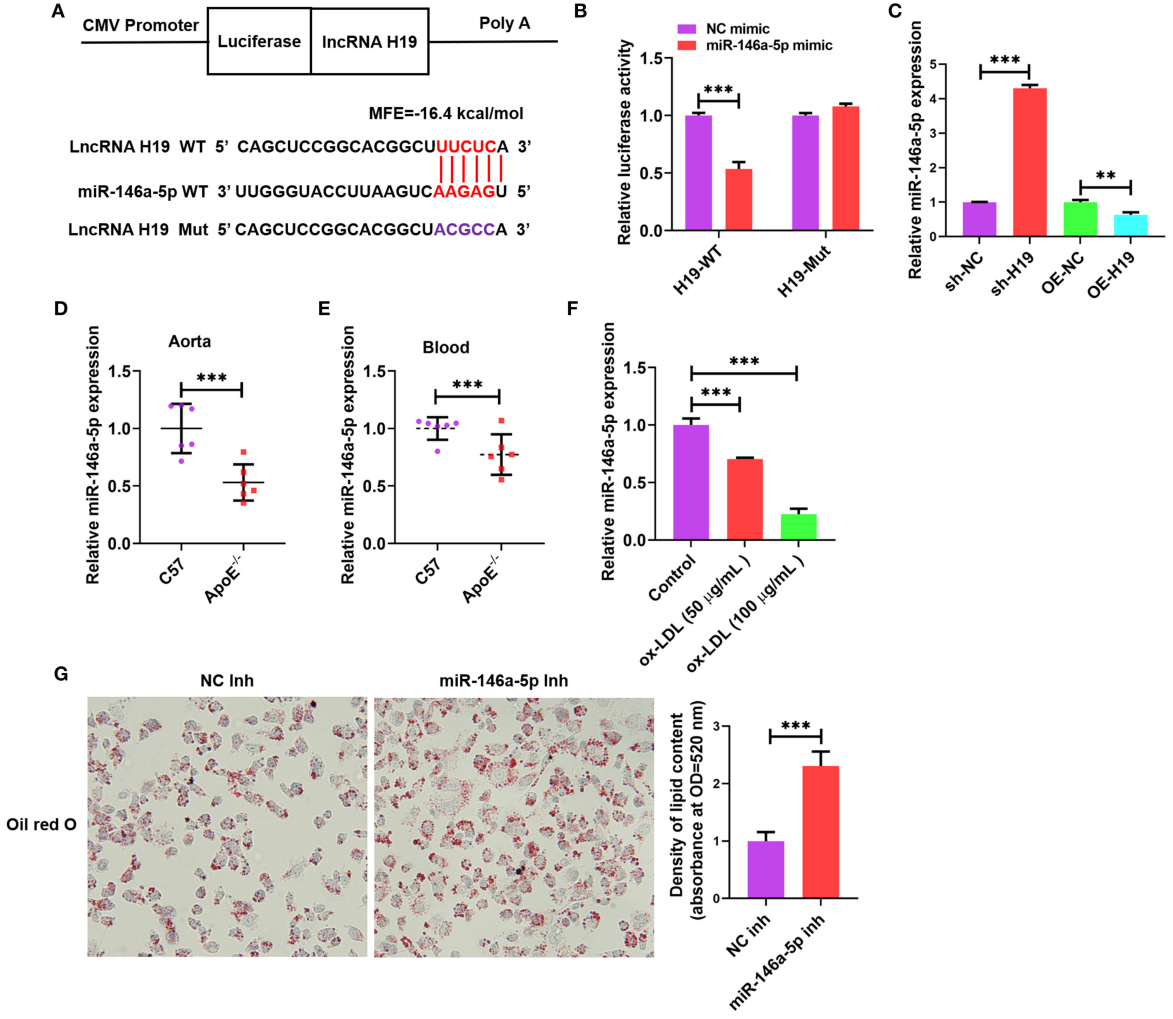
Validation of lncRNA H19 as a ceRNA for miR-146a-5p. **(A)** Schematic illustration of the miR-146a-5p binding site in the lncRNA H19 sequence and matching mutant. **(B)** The luciferase reporter plasmids lncRNA H19-WT or lncRNA H19-Mut were co-transfected into HEK-293T cells with miR-146a-5p mimic or its negative control for 48 h, ensued by the identification of luciferase activity. **(C)** THP-1 cells were transfected with sh-NC, sh-H19, OE-NC, or OE-H19 for 48 h. RT-PCR was carried out to identify miR-146a-5p expression. **(D,E)** C57BL/6 and apoE^−/−^ mice were supplied with a Western diet for 8 weeks (*n* = 6). miR-146a-5p expression in the blood and aorta were detected using RT-PCR. **(F)** THP-1 cells were treated with ox-LDL (100 μg/mL) or their controls for 24 h. The expression of miR-146a-5p was evaluated using RT-PCR. **(G)** THP-1 macrophages were transfected with NC inhibitor or miR-146a-5p inhibitor for 48 h, followed by oil red O staining. Data are represented as mean ± SD of 3 separate experiments. ^**^P < 0.01 and ^***^P < 0.001.

Cumulative evidence from research reports has demonstrated that lncRNAs can act as miRNA sponges, which interfere with miRNA at the post-transcriptional level thus decreasing the binding efficiency of miRNAs to target genes (34). We used an online bioinformatic dataset (BiBiServ2-RNAhybrid) (35) for identifying the potential miRNA recognition elements of lncRNA H19. We discovered that the lncRNA H19 sequence comprised one putative miR-146a-5p site in humans (Figure 4A). The sequences of lncRNA H19 together with the miR-146a-5p binding site and mutant lncRNA H19 were embedded downstream of the luciferase gene in the reporter plasmid to be used in luciferase assays. The results from the luciferase assay illustrated that transfection with miR-146a-5p mimic suppressed lncRNA H19 expression in the wild-type cohort as opposed to the control cohort, and this suppression was inverted after mutation of the binding site (Figure 4B), indicating the equivalent sequence-specific binding of miR-146a-5p to lncRNA H19. In addition, treating THP-1 cells with LV-lncRNA H19 (OE-H19) decreased miR-146a-5p levels (Figure 4C). However, the knockdown of lncRNA H19 (sh-H19) increased miR-146a-5p levels (Figure 4C). Therefore, lncRNA H19 functioned as a contending endogenous RNA (ceRNA) for miR-146a-5p.

As illustrated in Figures 4D,E, the miR-146a-5p expression was lower in the blood and aortic tissues of apoE^−/−^ mice supplied with Western diet as opposed to that in the wild-type mice. Furthermore, the miR-146a-5p levels were considerably decreased in ox-LDL-treated THP-1 cells as opposed to the control cells (Figure 4F). Transfection with miR-146a-5p inhibitor promoted the accumulation of lipids in ox-LDL-treated THP-1 cells (Figure 4G). These outcomes indicated that reduced miR-146a-5p expression aggravated atherosclerosis in apoE^−/−^ mice and also facilitated the accumulation of lipids in ox-LDL-treated THP-1 cells.

### ANGPTL4 Is a Target Gene of miR-146a-5p

**Figure 5:**
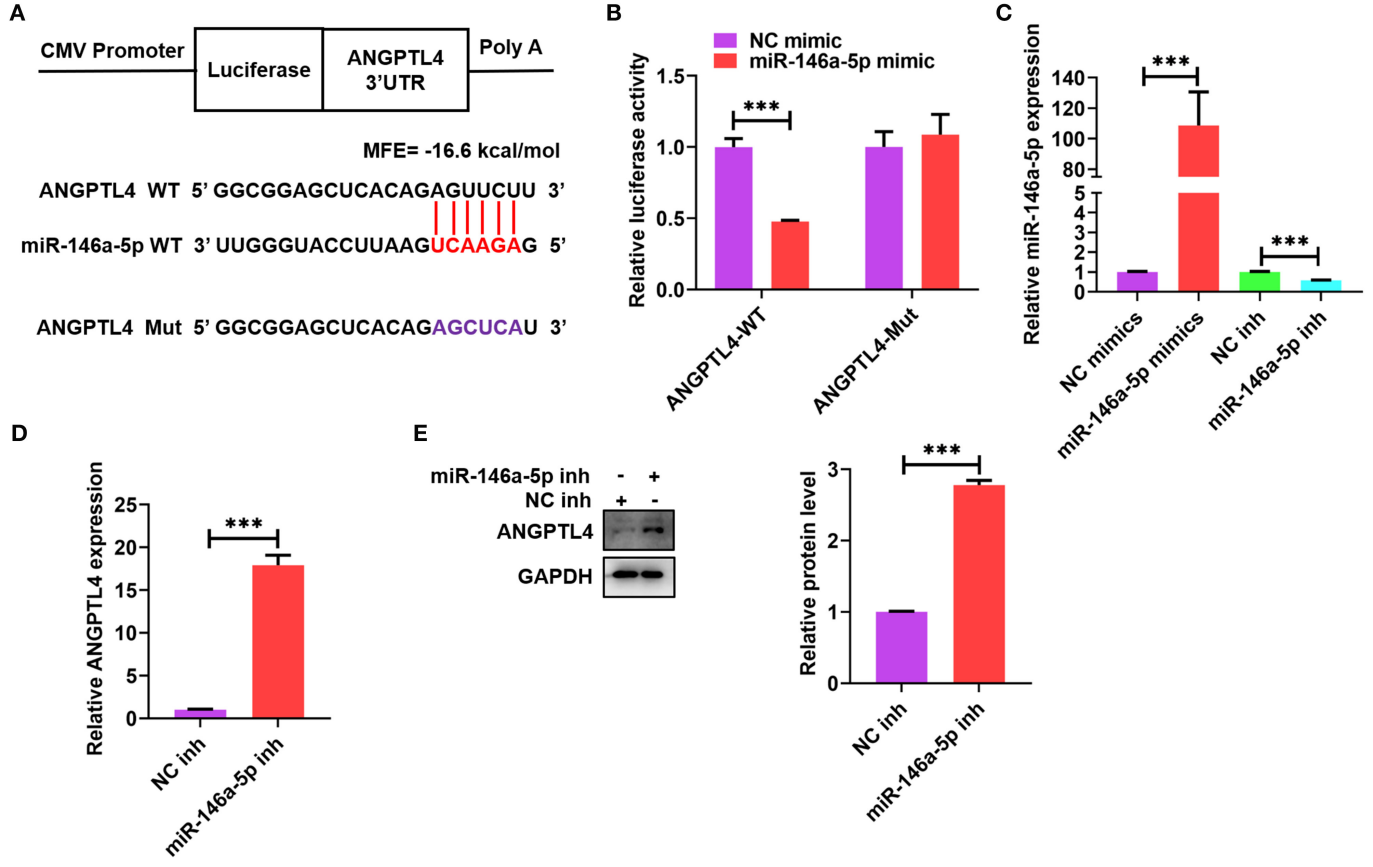
Identification of ANGPTL4 as a target gene of miR-146a-5p. **(A)** Schematic illustration of the miR-146a-5p binding site in the 3′-UTR of ANGPTL4 and the matching mutant. The mutated nucleotides were marked purple. **(B)** The luciferase reporter plasmids (ANGPTL4-WT and ANGPTL4-Mut) were co-transfected into HEK-293T cells with miR-146a-5p mimic or its control. The luciferase activity was assessed after a duration of 48 h. **(C–E)** THP-1 cells were transfected with miR-146a-5p mimic/inhibitor or their negative controls for 48 h.; **(C)** RT-PCR analysis of miR-146a-5p expression. **(D,E)** The mRNA and protein levels of ANGPTL4 were detected utilizing RT-PCR and WB, in that order. Data are articulated as mean ± SD of 3 separate experiments. ^***^P < 0.001.

Many studies have reported that ANGPTL4 is modulated by sponging miRNAs at the transcriptional level (36, 37). To identify prospective mRNAs that could be targeted by miR-146a-5p, we used a computational programming database (BiBiServ2-RNAhybrid) and found a putative binding site between ANGPTL4 3′-UTR and miR-146a-5p (Figure 5A). We subsequently built a luciferase reporter plasmid comprising a mutant (ANGPTL4-Mut) or wild-type (ANGPTL4-WT) miR-146a-5p binding site (Figure 5A). Transfection of these plasmids into HEK-293T cells together with miR-146a-5p mimic or mimic control was done to perform the luciferase reporter assay. Subsequently, the co-transfection of ANGPTL4-WT and miR-146a-5p mimic contributed to a considerable decline in the luciferase activity; however, this influence disappeared after the mutation of the miR-146a-5p binding site (Figure 5B). To ascertain whether miR-146a-5p might straightforwardly modulate the expression of ANGPTL4, THP-1 macrophages were transfected with miR-146a-5p mimic or inhibitor and the results indicated that miR-146a-5p mimic considerably reduced miR-146a-5p levels, whereas its inhibitor increased the levels, thus exhibiting an increased transfection efficiency (Figure 5C). Transfection with miR-146a-5p inhibitor was found to upregulate the protein and mRNA expression of ANGPTL4 (Figures 5D,E). These results revealed ANGPTL4 as a candid target of miR-146a-5p.

### ANGPTL4 Expression Was Sensitive to Lipids and Promoted Lipid Uptake in ox-LDL-Treated THP-1 Cells

**Figure 6:**
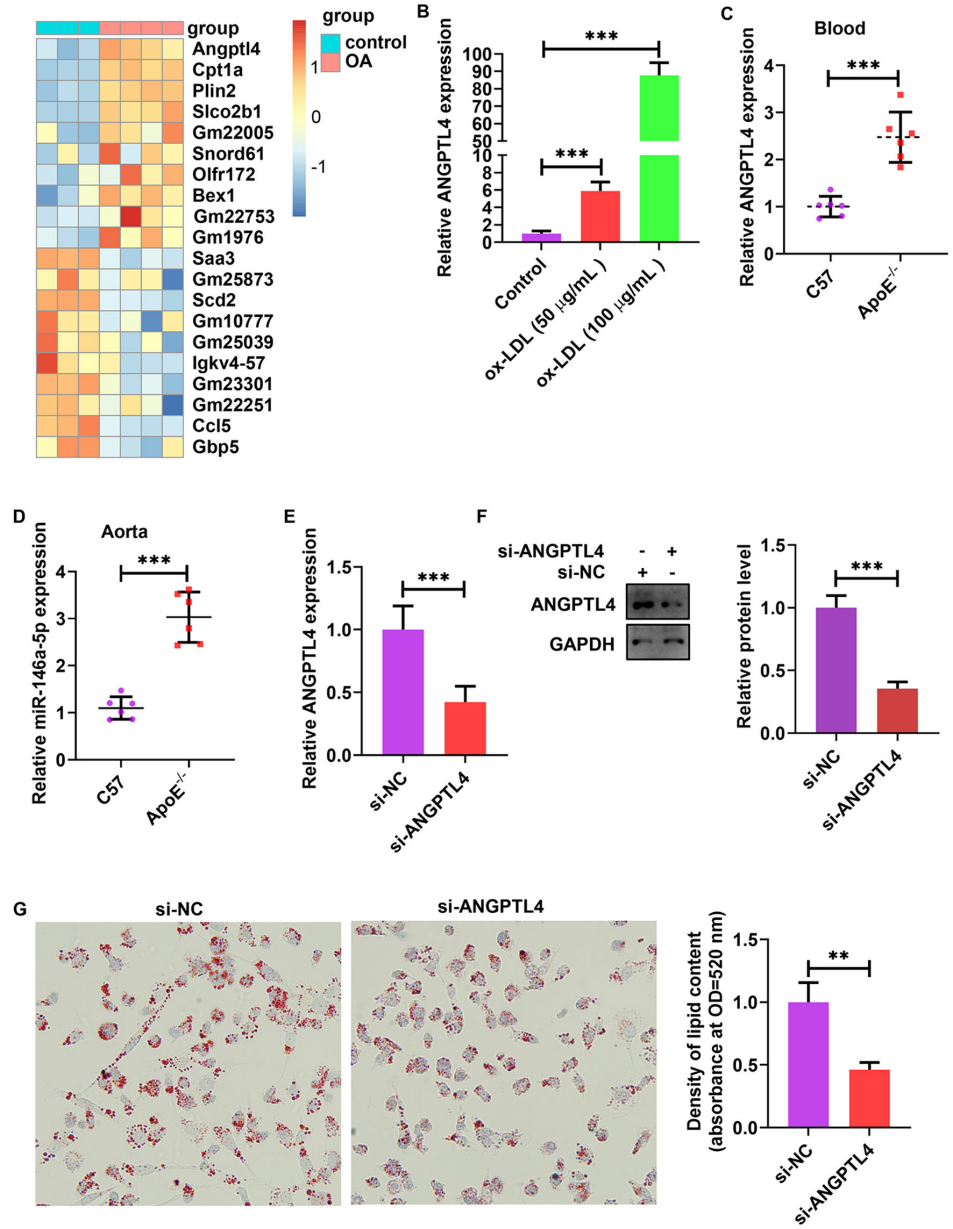
ANGPTL4 promoted lipid uptake in ox-LDL-treated THP-1 cells. **(A)** Heat map of 10 topmost downregulated and upregulated genes in BMDMs treated with oleic acid or control for 5 h. The data were obtained from a previously published dataset (GSE77104). **(B)** THP-1 cells were treated with ox-LDL (50 and 100 μg/mL) or their controls for 24 h. ANGPTL4 expression was evaluated using RT-PCR. **(C,D)** C57BL/6 and apoE^−/−^ mice were supplied with a Western diet for 8 weeks (*n* = 6). ANGPTL4 expression in the blood and aorta was detected using RT-PCR. **(E–G)** THP-1 cells were transfected with si-ANGPTL4 or its negative control for 48 h. **(E,F)** The protein and mRNA levels of ANGPTL4 were detected using RT-PCR and WB, successively. **(G)** Oil red O assay. Data are articulated as mean ± SD of 3 separate experiments. ^**^P < 0.01 and ^***^P < 0.001.

The analysis of publicly published datasets (GSE77104 and GSE83090) illustrated that ANGPTL4 is the highest induced gene in bone marrow-derived macrophages (BMDMs) and mouse peritoneal macrophages after treatment with oleic acid (OA) and acetylated (AC)-LDL (Figure 6A) (38–40). To scrutinize the function of endogenous ANGPTL4 in THP-1 cells, we first analyzed the regulation of ANGPTL4 expression. Treating THP-1 cells with ox-LDL markedly increased ANGPTL4 mRNA level (Figure 6B). ANGPTL4 expression was higher in the blood and aortic tissues of apoE^−/−^ mice fed a Western diet than that in the wild-type mice (Figures 6C,D). To examine the functional role of ANGPTL4 in lipid accumulation, we performed a loss-of-function study on THP-1 cells using ANGPTL4 siRNA. We found that the levels of expression of ANGPTL4 mRNA and protein were markedly repressed in THP-1 cells transfected with ANGPTL4 siRNA as opposed to the control cells (Figures 6E,F). Oil red O staining illustrated that ANGPTL4 knockdown reduced lipid accumulation in ox-LDL-treated THP-1 cells (Figure 6G). These results indicated that ANGPTL4 enhanced the accumulation of lipids in ox-LDL-treated THP-1 cells.

### lncRNA H19 Sponged miR-146a-5p to Increase ANGPTL4 Expression

**Figure 7:**
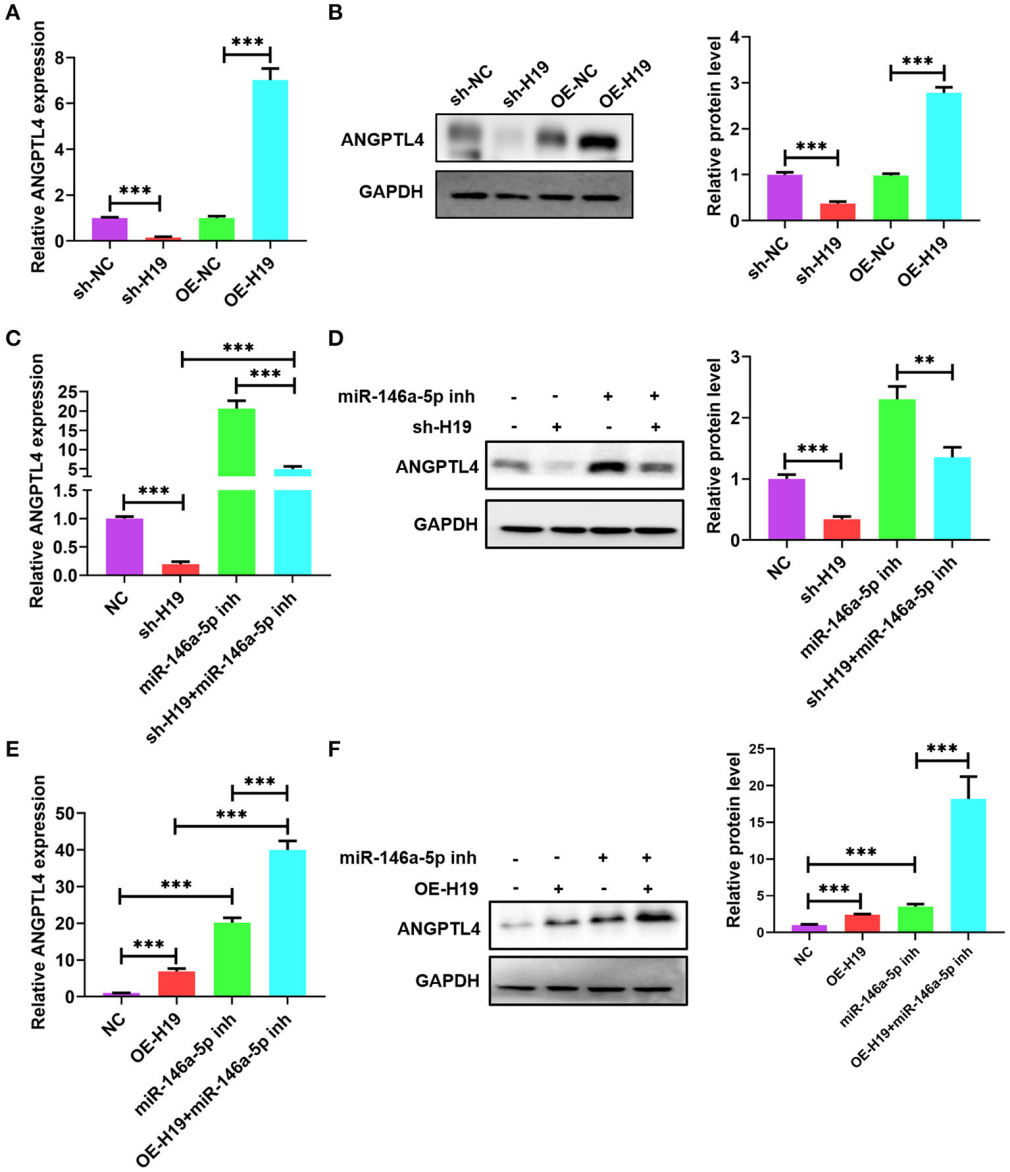
LncRNA H19 sponged miR-146a-5p to increase ANGPTL4 expression. **(A,B)** THP-1 cells were transfected with sh-NC, sh-H19, OE-NC, and OE-H19 for 48 h. The protein and mRNA levels of ANGPTL4 were detected utilizing RT-PCR and WB, successively. **(C,D)** THP-1 macrophages were transfected with miR-146a-5p inhibitor for 48 h and subsequently transduced with sh-H19 for 48 h (*n* = 3). The protein and mRNA levels of ANGPTL4 were measured utilizing RT-PCR and WB, successively. **(E,F)** THP-1 cells were transfected with miR-146a-5p inhibitor for 48 h and subsequently transduced with OE-H19 for 48 h (*n* = 3). The mRNA **(E)** and protein **(F)** levels of ANGPTL4 were determined utilizing RT-PCR and WB, in that order. Data were articulated as mean ± SD. ^**^P < 0.01 and ^***^P < 0.001 vs, control cohort.

Owing to the presence of a ceRNA network among lncRNA H19, ANGPTL4, and miR-146a-5p, we investigated whether miR-146a-5p is necessary for the regulatory impacts of lncRNA H19 on ANGPTL4 expression. The knockdown of lncRNA H19 decreased protein and ANGPTL4 mRNA levels; however, the overexpression of lncRNA H19 elevated the protein and mRNA levels of ANGPTL4 (Figures 7A,B). Co-transfection of THP-1 cells with miR-146a-5p inhibitor and sh-H19 was performed. As illustrated in Figures 7C,D, transfection with miR-146a-5p inhibitor considerably abrogated the effects of lncRNA H19 knockdown on ANGPTL4 expression. However, transfection with miR-146a-5p inhibitor enhanced the impact of lncRNA H19 overexpression on ANGPTL4 expression (Figures 7E,F). Therefore, we concluded that lncRNA H19 facilitated ANGPTL4 expression by serving as a ceRNA for miR-146a-5p.

## Discussion

Current research reports have illustrated that lncRNA H19 has a vital function in various cancers and CVDs (41–43); however, there is limited knowledge about its functions in the progression as well as the instability of atherosclerosis. In this research, we discovered that the levels of lncRNA H19 expression were considerably upregulated in mouse blood and aorta with atherosclerosis and ox-LDL-treated THP-1 macrophages, and the lncRNA H19 overexpression increased the atherosclerotic lesion size, promoted plaque lipid disposition and reduced collagen content in apoE^−/−^ mice, whereas a reverse impact was observed in response to knockdown of lncRNA H19. These results indicated that lncRNA H19 aggravates atherosclerosis.

Lipid accumulation is a critical step for lipid-laden foam cells formation, and a characteristic of atherosclerotic lesions throughout all stages of lipid-related diseases, such as fatty liver disease, obesity, myocardial infarction (MI), and atherosclerosis (11, 12). Our findings illustrated that the lncRNA H19 overexpression elevated lipid accumulation and its knockdown lowered lipid accumulation in THP-1-derived macrophages treated with ox-LDL, implying that lncRNA is important for lipid accumulation. The elevated lipid uptake and/or reduced cholesterol efflux results in the accumulation of lipid (44). Both 3-hydroxy-3-methylglutaryl-CoA reductase (HMGCR) and ABCA1 are responsible for lipid biosynthesis and cholesterol efflux by macrophages (45–47). ANGPTL4 is the main upregulated gene in macrophage-derived foam cells in atherosclerotic plaques as evidenced by microarray analysis and RT-PCR assays (40). Macrophages isolated from LDLR^−/−^ mice significantly upregulated ABCA1 expression and downregulated HMGCR expression as compared with the wild-type mice, indicating excessive lipid accumulation in macrophages (40). Furthermore, ANGPTL4-deficient mice (ANGPTL4^−/−^ LDLR^−/−^ mice) exhibited markedly reduced atherosclerotic lesions as compared with LDLR^−/−^ mice and significantly decreased macrophage accumulation (40), implying that ANGPTL4 plays a critical function in lipid accumulation. In addition, ANGPTL4-deficient mice (ANGPTL4^−/−^ apoE^−/−^ mice) exhibited decreased triglyceride levels and atherosclerotic lesion size as opposed to apoE^−/−^ANGPTL 4^+/+^ mice (48). The peritoneal macrophages, obtained from the ANGPTL 4^−/−^ mice, exhibited a considerable reduction in cholesteryl ester (CE) accumulation as opposed to the ANGPTL 4^+/+^ mice (48). We reported that ANGPTL4 knockdown by siRNA decreased the accumulation of lipids in THP-1-derived macrophages under ox-LDL stimulation. This suggests that ANGPTL4 inhibits lipid accumulation. An additional analysis illustrated that the elevated ANGPTL4 expression *via* the overexpression of lncRNA H19 promoted lipid accumulation in macrophages. Therefore, the prevention of ANGPTL4-mediated lipid accumulation is an important mechanism by which lncRNA H19 facilitates lipid accumulation and aggravates atherosclerosis.

LncRNAs have been known to be key modulators of different biological activities, and their aberrant expression is strongly linked to the occurrence and development of several illnesses (49–51). Contemporary research reports have demonstrated that lncRNAs including KCNQ1OT1, CHROME, and lnc-HC participate in lipid metabolism, macrophage cholesterol efflux, and lipid accumulation (9, 52, 53), which have critical regulatory functions in the development of atherosclerotic diseases (10, 11). However, a few studies have demonstrated the functional roles of lncRNAs in atherosclerotic diseases. Atherosclerotic diseases have been identified as the major contributor to morbidity and fatality globally (54). Thus, it is imperative to find possible biomarkers and treatment targets against atherosclerotic diseases.

In this study, by comparing the aorta and blood of apoE^−/−^ and C57BL/6 mice fed HFD, we identified that lncRNA H19 could serve an essential function in atherosclerosis. Consistent with our findings, contemporary research reports have demonstrated that lncRNA H19 was upregulated in the blood sample of atherosclerosis patients (14, 25), suggesting that lncRNA H19 is a potential biomarker and prognostic indicator for atherosclerotic disease. Moreover, utilizing RNA-FISH analysis, we verified that lncRNA H19 was mainly localized in the macrophages of atherosclerotic plaques. These findings indicated suggested that elevated expression of lncRNA H19 significantly aggravated the progression and instability of atherosclerosis. Owing to a lack of clinical studies, which may provide reliable clinical data, it is difficult to adequately determine whether lncRNA H19 is linked to prognosis or might be used as a novel biomarker for vulnerable plaques.

Contemporary studies have demonstrated that lncRNA H19 deficiency alleviated macrophage activation, lipid accumulation, and inflammation *in vivo* (55, 56). To examine the role of lncRNA H19 and evaluate its treatment prospective in atherosclerosis, we overexpressed or knocked down lncRNA H19 *in vivo* using AAV in apoE^−/−^ mice. The knockdown of lncRNA H19 significantly alleviated the atherosclerotic plaque area *in vivo*; however, the overexpression of lncRNA H19 enlarged the plaque area. ANGPTL4 has been closely associated with lipid metabolism (57). In this research, by conducting *in vitro* loss-of-function and gain-of-function experiments in THP-1 cells, we demonstrated that lncRNA H19 positively regulated the expression of ANGPTL4 in a transcription-independent way. Therefore, these results reveal that ANGPTL4 may be a downstream target of lncRNA H19.

Current studies have reported that lncRNAs situated in both the nucleus and cytoplasm of cells and the subcellular localization of lncRNAs offer a novel perspective for understanding their function and support the hypothesis for the underlying molecular mechanism (58, 59). Based on this hypothesis, lncRNAs bind to miRNAs and modulate the expression of miRNA-targeted genes. Foremost, by conducting RNA-FISH assays, we discovered that lncRNA H19 was localized in the cytoplasm of THP-1 cells, and its levels in cytoplasm elevated after ox-LDL treatment. The outcomes illustrated that lncRNA H19 participated in the post-transcriptional modulation in cytoplasm. Recent evidence from research reports has verified that lncRNAs serve as miRNA sponges and regulate the de-repression of miRNA targets (34, 60). Furthermore, we forecasted the interplay between miR-146a-5p and lncRNA H19 utilizing an online bioinformatic dataset and discovered that lncRNA H19 encompasses a miR-146a-5p target site. Additionally, ANGPTL4 was found to be a target gene of miR-146a-5p. lncRNA H19 knockdown decreased ANGPTL4 levels, and this effect was reversed following the transfection with miR-146a-5p inhibitor. Thus, we suggested that lncRNA H19 served as a ceRNA to modulate ANGPTL4 expression by sponging miR-146a-5p in THP-1 cells, which could be one of the fundamental mechanisms through which lncRNA H19 functions as a crucial regulator of lipid accumulation and formation of atherosclerotic plaque.

The surfacing of atherosclerosis-associated lncRNAs as significant modulators of gene expression has considerably transformed our comprehension of the basic pathological mechanisms of atherosclerosis. In conclusion, this research depicted a pathogenic function of lncRNA H19 in promoting the development of atherosclerosis and revealed a new mechanism for ANGPTL4 modulation. lncRNA H19 interacts with miR-146a-5p to upregulate the expression of ANGPTL4 by serving as a ceRNA, which contributes to the promotion of lipid accumulation and atherosclerotic plaque formation. Therefore, the inhibition of lncRNA H19 expression could be an auspicious approach for preventing and treating atherosclerotic CVD.

## Data Availability Statement

The original contributions presented in the study are included in the article/supplementary material, further inquiries can be directed to the corresponding author.

## Ethics Statement

The animal study was reviewed and approved by the Sixth Affiliated Hospital of Guangzhou Medical University.

## Author Contributions

W-CY and S-FH devised and made an outline of the article. W-CY, S-FH, and GZ reviewed the literature and scripted the manuscript. W-CY, S-FH, GZ, and X-FP reviewed the literature and offered important recommendations. W-CY contributed ideas and provided the preliminary design. All the authors have given their approval for the submission of this manuscript and made pertinent contributions to the inception, design, and revision of this article.

## Funding

The authors appreciatively recognize the funding support received from the Medical Research Fund project of Qingyuan People's Hospital (20190221, 20190219, and 20190226), the Guangdong Natural Sciences Foundation (2019A1515110080), the Guangdong Medical Research Foundation (B2021153), and the National Natural Sciences Foundation of China (81901972 and 82000407).

## Conflict of Interest

The authors declare that the research was conducted in the absence of any commercial or financial relationships that could be construed as a potential conflict of interest.

## Publisher's Note

All claims expressed in this article are solely those of the authors and do not necessarily represent those of their affiliated organizations, or those of the publisher, the editors and the reviewers. Any product that may be evaluated in this article, or claim that may be made by its manufacturer, is not guaranteed or endorsed by the publisher.
